# Correction: Transient changes in white matter microstructure during general anesthesia

**DOI:** 10.1371/journal.pone.0250449

**Published:** 2021-04-15

**Authors:** The PLOS ONE Staff

## Notice of Republication

This article was republished on April 6, 2021, to correct the second author’s affiliations and contributions, which were erroneously left blank in the originally published version of the article. The publisher apologizes for the errors. Please download this article again to view the correct version. The originally published, uncorrected article and the republished, corrected articles are provided here for reference.

## Supporting information

S1 File Originally published, uncorrected article. (PDF)

S2 File Republished, corrected article. (PDF)
