# Supplementary material for: Correction: Transient changes in white matter microstructure during general anesthesia
Source: PLoS One. 2021 Apr 15;16(4):e0250449. doi: 10.1371/journal.pone.0250449 (PMC8049249; doi:10.1371/journal.pone.0250449)
Supplement: S1 File — (PDF) [file pone.0250449.s001.pdf]

PLOS ONE

RESEARCH ARTICLE

Transient changes in white matter microstructure during general anesthesia

Cheuk Y. Tang[1,2,3]*, Victoria X. Wang, Min Yin Lun[1], Joshua S. Mincer[4], Johnny C. Ng[1], Jess W. Brallier[4], Arthur E. Schwartz[5], Helen Ahn[5], Patrick J. McCormick[4], Tommer Nir[5], Bradley Delman[1], Mary Sano[4], Stacie G. Deiner[6], Mark G. Baxter[2]

1 BioMedical Engineering Imaging Institute, Icahn School of Medicine at Mount Sinai, New York, NY, United States of America, **2** Nash Family Department of Neuroscience, Icahn School of Medicine at Mount Sinai, New York, NY, United States of America, **3** Department of Psychiatry, Icahn School of Medicine at Mount Sinai, New York, NY, United States of America, **4** Department of Anesthesiology and Critical Care Medicine, Memorial Sloan Kettering Cancer, New York, NY, United States of America, **5** Department of Anesthesiology, Perioperative and Pain Medicine, Icahn School of Medicine at Mount Sinai, New York, NY, United States of America, **6** Department of Anesthesiology, Dartmouth Hitchcock, Lebanon, NH, United States of America

* Cheuk.Tang1@gmail.com

Check for updates

OPEN ACCESS

Citation: Tang CY, Wang VX, Lun MY, Mincer JS, Ng JC, Brallier JW, et al. (2021) Transient changes in white matter microstructure during general anesthesia. PLoS ONE 16(3): e0247678. https://doi.org/10.1371/journal.pone.0247678

Editor: Quan Jiang, Henry Ford Health System, UNITED STATES

Received: September 25, 2020

Accepted: February 10, 2021

Published: March 26, 2021

Copyright: © 2021 Tang et al. This is an open access article distributed under the terms of the Creative Commons Attribution License, which permits unrestricted use, distribution, and reproduction in any medium, provided the original author and source are credited.

Data Availability Statement: The publicly accessible data is available through DRYAD, via this link: https://datadryad.org/stash/share/urpv1JLCCuHKNOUF_yr3LGTGENPl7chhjSAY_1ty3YQ.

Funding: This study was funded by: NIH 5R01AG046634, Trajectory of Recovery in the Elderly, PI: Mark Baxter (MB) The funders had no role in study design, data collection and analysis, decision to publish, or preparation of the manuscript.

Abstract

Cognitive dysfunction after surgery under general anesthesia is a well-recognized clinical phenomenon in the elderly. Physiological effects of various anesthetic agents have been studied at length. Very little is known about potential effects of anesthesia on brain structure. In this study we used Diffusion Tensor Imaging to compare the white matter microstructure of healthy control subjects under sevoflurane anesthesia with their awake state. Fractional Anisotropy, a white mater integrity index, transiently decreases throughout the brain during sevoflurane anesthesia and then returns back to baseline. Other DTI metrics such as mean diffusivity, axial diffusivity and radial diffusivity were increased under sevoflurane anesthesia. Although DTI metrics are age dependent, the transient changes due to sevoflurane were independent of age and sex. Volumetric analysis shows various white matter volumes decreased whereas some gray matter volumes increased during sevoflurane anesthesia. These results suggest that sevoflurane anesthesia has a significant, but transient, effect on white matter microstructure. In spite of the transient effects of sevoflurane anesthesia there were no measurable effects on brain white matter as determined by the DTI metrics at 2 days and 7 days following anesthesia. The role of white matter in the loss of consciousness under anesthesia will need to be studied and MRI studies with subjects under anesthesia will need to take these results into account.

Introduction

Cognitive dysfunction in elderly patients after they undergo surgery under general anesthesia is a well-known phenomenon, but it is unclear whether this is caused by the surgical procedures or the anesthesia. Little is known about how general anesthesia causes a reduction in nerve transmission and subsequent loss of consciousness and the effects of general anesthesia

Competing interests: The authors have declared that no competing interests exist.

Abbreviations: AD, Axial Diffusivity; ADC, Apparent Diffusion Coefficient; AQP4, Aquaporin-4; BOLD, Blood Oxygenation Level Dependent; CSF, Cerebral Spinal Fluid; DTI, Diffusion Tensor Imaging; FA, Fractional Anisotropy; fDTI, functional Diffusion Tensor Imaging; ICP, Intracranial Pressure; ISF, Interstitial Space Fluid; MD, Mean diffusivity; RD, Radial Diffusivity; ROI, region of Interest; TBSS, Tract Based Spatial Statistics.

on brain structure has not been studied before. Some neuroimaging studies necessitate the use of anesthesia. Examples are patients that cannot stay still long enough to complete an imaging protocol, certain pediatric populations, patients on ventilators, patients with claustrophobia or other complicating factors [1–3]. Another situation where patients are scanned under anesthesia is in intraoperative MRI procedures [4]. We collected these data in the context of the TORIE (Trajectory of Recovery in the Elderly) study [5] which was designed to investigate the recovery of cognitive function after general anesthesia without surgery in healthy adults 40–80 years old, spanning the age range of individuals that are at elevated risk of postoperative neurocognitive disorders including Postoperative Delirium (PD) and Postoperative Cognitive Dysfunction (POCD) [6, 7]. In this study we sought to analyze the effects of general anesthesia on brain white matter.

Materials and methods

Subjects

This analysis is part of a larger study on the effects of anesthesia on cognitive function in the elderly in the absence of surgery (Trajectory of Recovery in the Elderly [TORIE] (Trajectory of Recovery in the Elderly), NIH 1R01AG046634, clinicaltrials.gov registration NCT 2275026). The full protocol for this study is published [5]. This study was approved by the Institutional Review Board of the Icahn School of Medicine at Mount Sinai (New York, NY, USA; IRB@mssm.edu, 212-824-8200). Participants were recruited through local contacts and IRB-approved advertisements in local media and online. Potential participants were pre-screened by telephone by both research staff and a study anesthesiologist. Informed written consent was obtained by participants at the first in-person visit. Specific inclusion criteria were adults aged 40–80, American Society of Anesthesiologists (ASA) Physical Status 1 (no medical comorbidities) or 2 (one or more medical comorbidities which do not impact the patient's function), and no underlying cognitive dysfunction as determined from baseline cognitive testing before general anesthesia. Exclusion criteria included contraindication to MRI scanning (implanted metal, presence of tattoos, claustrophobia), current smoking, use of illicit drugs, excessive use of alcohol, or other diseases that could affect response to anesthesia or alter brain physiology. Participants were excluded after recruitment and consent if the scan prior to anesthesia revealed any of the following: cerebral microvascular disease, any mass, evidence of old infarct (even without clinical signs), atrophy and/or ventriculomegaly greater than expected for age in the neuroradiologist's judgment. Age-appropriate changes, such as mild cortical atrophy, were not grounds for exclusion. Participants were also excluded if baseline neuropsychological testing suggested poor or abnormal baseline cognitive function in the judgment of the study neuropsychologist. Sixty-eight healthy participants were analyzed in this study with an average age of 58.6 years (30F/38M). Although not used in the current analysis, participants received a battery of cognitive tests including the Postoperative Quality of Recovery Scale (PQRS) and NIH Toolbox Cognitive Battery to assess executive function, attention, episodic memory, working memory and processing speed.

Imaging

In the TORIE imaging protocol scans were acquired at 5 different time points: before induction of anesthesia (***PRE***), twice (at 40 mins (***A1***) and 100 mins (***A2***) post induction) during a 2 hour general anesthesia at a depth of one age-adjusted MAC (minimum alveolar concentration) of sevoflurane, 1 day post anesthesia (***D1***) and 7 days post anesthesia (***D7***). All scans were performed on a Siemens 3T Skyra using a 32 channel head coil. Sequences included Dual Echo TSE (PD-T2), GE-EPI BOLD N-Back, GE-EPI ME-BOLD Resting State, Diffusion Tensor

Imaging (DTI), T1 3D MP-RAGE, T2/FLAIR, pulsed arterial spin labeling (PASL), and Susceptibility Weighted Imaging (SWI). For this study only the DTI and anatomical T1's were analyzed. T1 MP-RAGE was performed with the following protocol: T1-weighted anatomical images will be acquired with an MPRAGE sequence (FOV 256 × 256 × 176 mm, 0.8 mm isotropic resolution, TR/TE/TI = 2400/3.2/1000ms, bandwidth 280 Hz/Pixel, echo spacing 7.6 ms, in-plane acceleration factor 2, and total acquisition time ~ 7 min). DTI: diffusion MRI data were acquired with a MB accelerated single shot spin echo EPI sequence (FOV 208 × 176 mm, matrix 114 × 96, slice thickness 1.8 mm, 72 slices for whole brain coverage, TR/TE = 3650/85 ms, Stejskal-Tanner (i.e., monopolar) diffusion encoding with diffusion G_{max} ~ 43mT/m, phase partial Fourier 6/8, MB factor 3, blipped CAIPIRINHA phase-encoding shift = FOV/3, bandwidth ~ 1700 Hz/Pixel, echo spacing ~0.7 ms, diffusion encoding directions 64 with 4 non-diffusion weighted (i.e., b_0) images, b value 1250 s/mm^2, total acquisition time ~ 12 min, with two phase-encoding direction reversed averages to correct eddy current distortion and improve signal-to-noise ratio (SNR).

Anesthesia

The first day of imaging included the pre-anesthesia scan and the scans during anesthesia. Anesthesia staff performed a preanesthesia evaluation and confirmed the participant's eligibility. The imaging suite has a complete anesthesia setup, including an MRI compatible anesthesia machine and a set of vital sign monitors (blood pressure, ECG, oximetry, end-tidal CO2 and gas analysis). Following the pre-anesthesia scans, the MRI bed was moved out of the scanner and the anesthesia induced using propofol (approximately 2 mg/kg) followed by the insertion of a laryngeal mask airway (LMA). Anesthesia was maintained using sevoflurane. Anesthetic depth during the transition between propofol and sevoflurane was adjusted to a bispectral index between 40 and 60 using a Bispectral Index Monitor (Covidien, MA, USA) which was removed for subsequent scanning. Participants resumed spontaneous respiration under anesthesia. This induction procedure lasted about 15 minutes. The participant was then moved back into the scanner and imaged under anesthesia for about 2 hours. After the last scan, the subject emerged from anesthesia and the LMA was removed. After it was determined that they were sufficiently conscious to perform a functional task, which took about 15 minutes on average, the scanner bed was moved back into the scanner bore for the post anesthesia scans. Participants were reassessed using the PQRS battery and transported to a postanesthesia care unit (PACU). Follow up scans without anesthesia was performed on the next day and again 7 days later.

Analysis

In this analysis we focused on diffusion tensor metrics. Diffusion Tensor Images are eddy-current-corrected and fractional anisotropy (FA), Radial Diffusivity (RD), Axial Diffusivity (AD) as well as mean diffusivity maps (MD) were calculated using FSL (www.fmrib.ox.ac.uk/fsl). Exploratory whole brain group comparisons of the diffusion parameters are performed. FA (and other DTI metrics) images were spatially normalized to the ICBM template using Tract Based Spatial Statistics (TBSS) [8]. The procedure involves a skeletonization of the FA images to obtain centers of white matter tracts. Voxel-wise statistics are performed only on the white matter skeleton in order to reduce the chance of type I errors due to imperfections in normalization. The parameters used to warp the FA images to ICBM template to the white matter skeleton were applied to the MD, RD and AD images for statistical comparisons. Randomise is an FSL routine for permutation based inference testing that is used for voxel-wise general linear modeling to test for differences between the conditions. Clusters are identified using the TFCE

(Threshold-Free Cluster Enhancement) that is optimized for permutation (n = 5000) based inference testing of skeletonized images [9]. Age and sex were used as covariates. We separated into 5 conditions per subject: 1 before anesthesia induction, 2 during anesthesia (A1 (at approximately 40 mins), A2 (at approximately 100 mins)), 1 at 1 day follow up and 1 at 7 day follow up. Whole brain as well as individual tracts' DTI metrics were computed using the Johns Hopkins University ICBM-DTI-81 white matter atlas: mean values were calculated based on the average voxel intensities averaged over all the regions of interests of the atlas. Statistical analysis of individual tracts was performed using Statistica V13 (Statsoft Inc., Tulsa, OK).

T1-MPRAGE images were processed through Freesurfer. Cortical reconstruction and volumetric segmentation was performed using the standard recon-all pipeline of the Freesurfer image analysis software (version 5.3.0), which is documented and freely available online (http://surfer.nmr.mgh.harvard.edu). Regions of interest (ROIs) were labeled using an automatic labeling system [10]. Processing included motion correction, removal of non-brain tissue, automated Talairach transformation, segmentation of the subcortical regions and deep gray matter structures intensity normalization, tessellation of the gray matter-white matter boundary, automated topology correction, and surface deformation following intensity gradients. Gray matter volume, white matter volume, and cortical thickness measures were computed.

Statistical methods

All voxel by voxel wise statistics were performed using FSL as described above. ROI and whole brain based analysis were performed using Statistica V13. All time point comparisons were performed using a paired t-test and corrected for age and sex. Pearson correlation analyses were performed between whole brain DTI metrics and age. Multiple linear regression was used to test for effect of age and sex on the change in DTI metrics.

Results

Whole brain voxel-based analyses using TBSS showed that Fractional Anisotropy (FA) was decreased throughout the brain during anesthesia when compared to the awake state before anesthesia. On the other hand, MD, RD and AD were all increased during anesthesia (Fig 1). Although AD had slightly fewer significant voxels than RD. Statistical analysis on whole brain white matter DTI metrics showed that FA was decreased while MD, AD and RD were increased during anesthesia when compared to time points before or after the administration of anesthesia. In addition, FA was lower and MD, AD and RD were higher at 100 min than at 40 min post administration of anesthesia. There were no differences between pre-anesthesia and day 1 or day 7 post anesthesia (Fig 2). These significances were corrected for age and sex. Table 1 shows the magnitude of the white matter mean values and the percent changes between the time points.

At baseline, linear correlation analysis showed that whole brain white matter FA had a significant negative correlation with age ($p = 1.09e-5$, $R\text{-}sqr = 0.256$) while MD ($p = 4.07e-9$, $R\text{-}sqr = 0.410$), AD ($p = 5.781e-8$, $R\text{-}sqr = 0.362$) and RD ($p = 6.269e-9$, $R\text{-}sqr = 0.403$) were positively correlated (Fig 3A). To visualize age differences we split up the data into 4 age groups [40–49 (n = 19), 50–59 (n = 18), 60–69 (n = 13), and 70–80 (n = 18)] as per the main protocol [5]. Group means of the DTI metrics are shown in Fig 3B. Multiple linear regression was used to investigate the relationship between change in FA, calculated as the value at 40 minutes after drug administration minus the baseline value, with age and sex. Baseline FA was included as a covariate in the model. Adjusting for sex and baseline FA, there was no significant association between change in FA and age ($\beta = 0.000003$; 95% CI: -.000098 to 0.000104; $p = 0.95$).

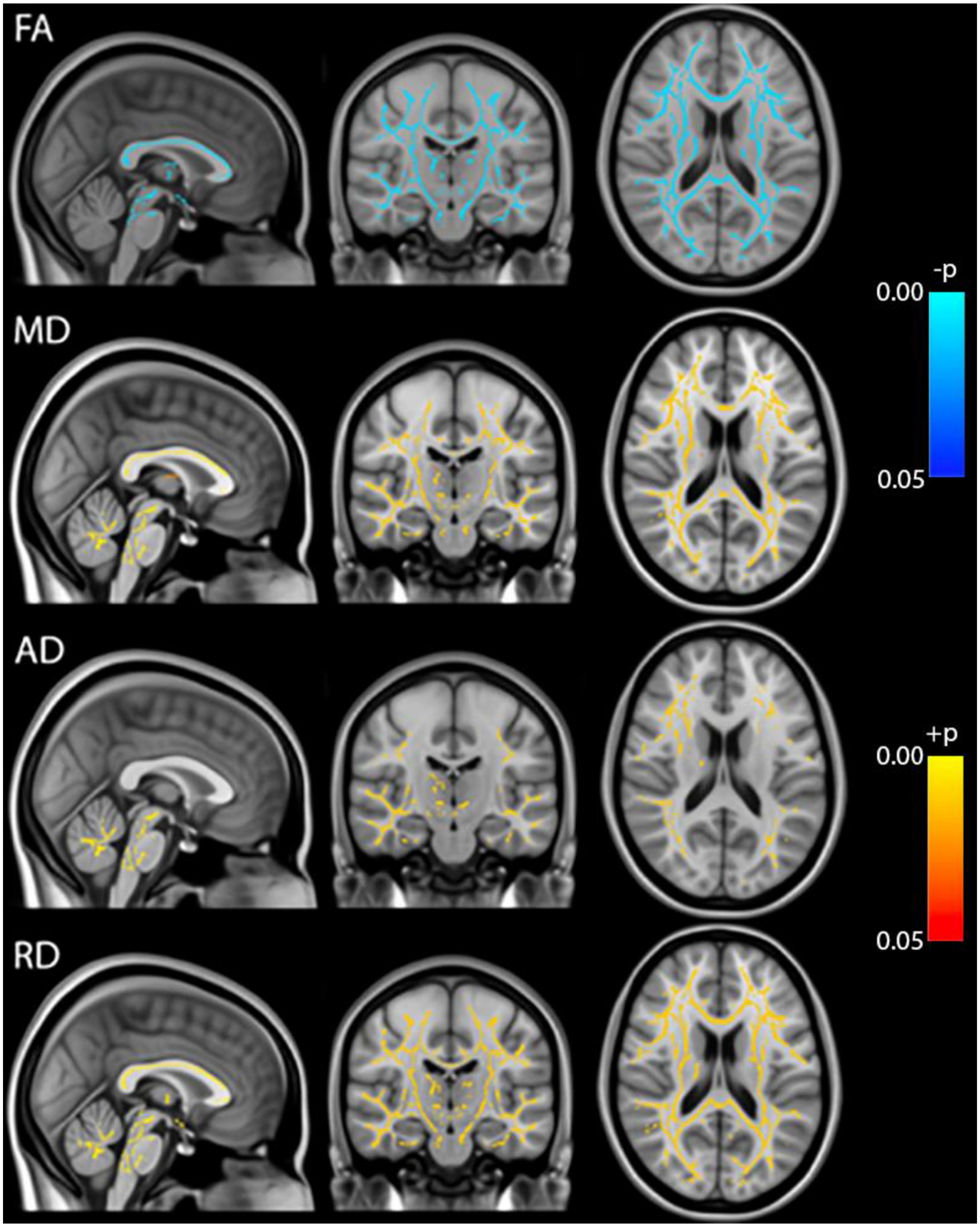

Fig 1. TBSS analysis of DTI metrics (FA, MD, AD, RD). Blue: Pre-anesthesia > during anesthesia, Red: Pre-anesthesia < during anesthesia, p<0.05 FWE-corrected.

https://doi.org/10.1371/journal.pone.0247678.g001

Similarly, there was no significant association between change in FA and sex (β = -0.000178; 95% CI: -.002184 to 0.001827; p = 0.86) after controlling for age and baseline FA. No significance associations were found between changes in MD, AD and RD and age or sex (Tables 2 and 3).

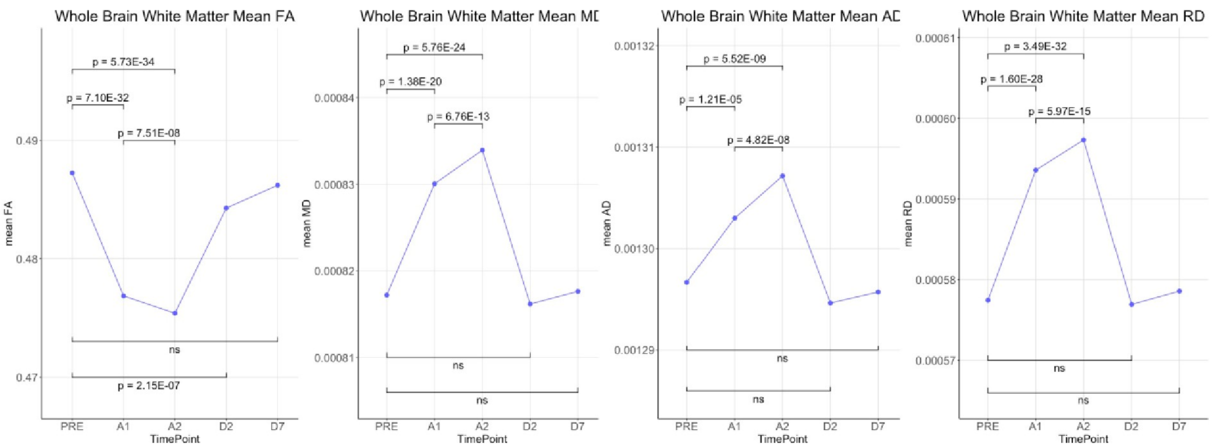

Fig 2. Statistical comparisons of whole brain means of DTI metrics (FA, MD, AD and RD) over the 5 time-points.

https://doi.org/10.1371/journal.pone.0247678.g002

The 48 ROIs from the Johns Hopkins white matter atlas showed the same significances and directions except for two regions near the inferior cerebellar peduncles (not significant). The other 46 white matter tracts all had significances between $p<10^{-5}$ and $p<10^{-31}$ after correction for age and sex (Table 4).

Analysis of the structural MRI (T1-MPRage) using Freesurfer showed that both left and right hemispheric white matter volumes were smaller during anesthesia ($p<0.018$ and $p<0.008$ respectively). Out of the 68 white matter regions extracted, 21 were smaller and 2 (left and right entorhinal cortex) were larger during anesthesia. Out of the 68 gray matter regions, 42 regions were larger under anesthesia while 2 (left and right frontal poles) were smaller (Table 5).

Analysis of ventricular volume showed that overall CSF volume was increased during anesthesia. Individual ventricles showed that the 3rd ventricle was increased while the 4th ventricle was decreased. Lateral ventricle volumes were also increased but were not significant (Table 6).

Discussion

The source of Diffusion Weighted signal based on the acquisition sequences used in this study is believed to be most sensitive to extracellular water [11, 12]. FA measures the coherence of

Table 1. Top: Whole brain white matter means of DTI metrics (FA, MD, AD and RD) for the 5 time points. Bottom: % change between the time points (* indicates statistical significance). **PRE**—before induction of anesthesia, **A1**–40 mins, **A2**–100 mins after induction of anesthesia, **D2** – 24hrs after general anesthesia, **D7**–7 days after general anesthesia.

Time point	FA	MD (mm^2/s)	AD (mm^2/s)	RD (mm^2/s)
**PRE**	0.487	0.000817	0.001297	0.000577
**A1**	0.477	0.000830	0.001303	0.000594
**A2**	0.475	0.000834	0.001307	0.000597
**D2**	0.484	0.000816	0.001295	0.000577
**D7**	0.486	0.000818	0.001296	0.000579
**% change (A1-Pre)**	-2.13 *	1.57 *	0.49 *	2.79 *
**% change (A2-Pre)**	-2.43 *	2.05 *	0.81 *	3.44 *
**% change (A2-A1)**	-0.31 *	0.47 *	0.32 *	0.63 *
**% change (D2-Pre)**	-0.61 *	-0.13	-0.16	-0.09
**% change (D7-pre)**	-0.21	0.05	-0.07	0.19

https://doi.org/10.1371/journal.pone.0247678.t001

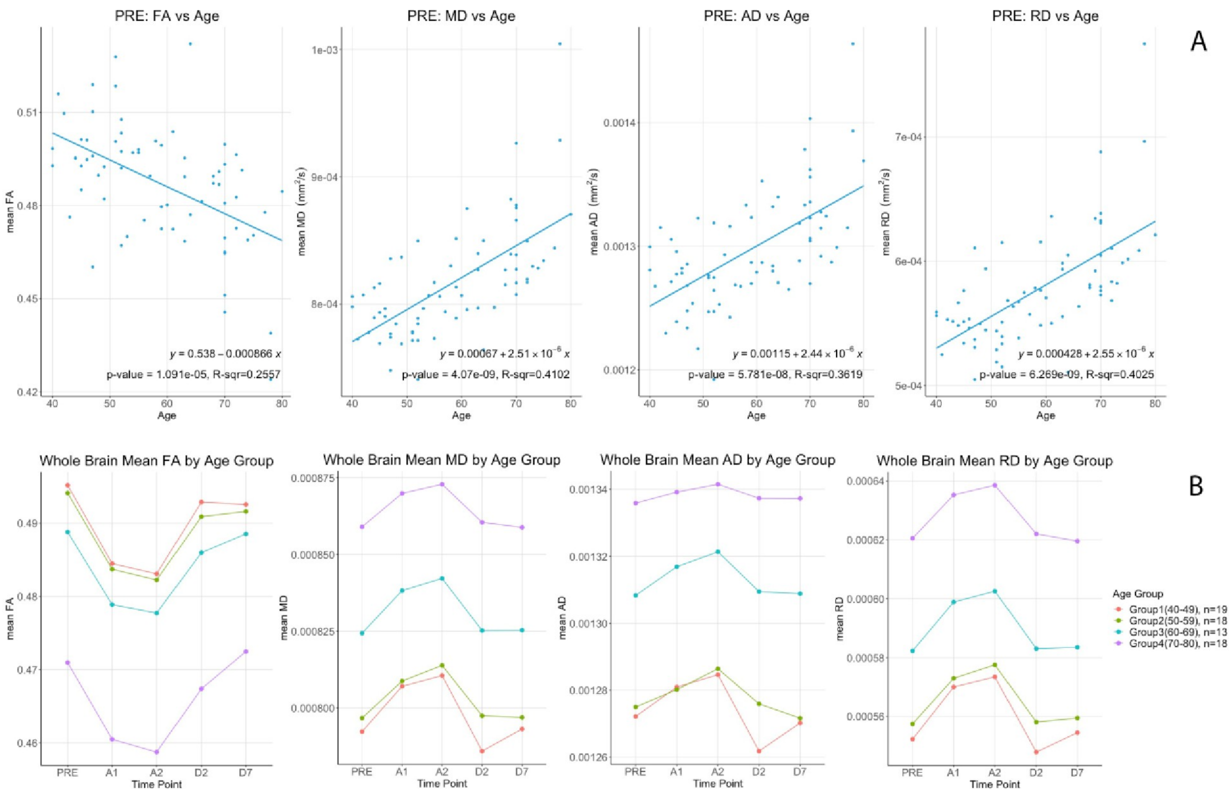

Fig 3. Top (A) DTI metrics (FA, MD, AD & RD) correlations versus age at baseline (PRE). Bottom (B) Differential changes of (FA, MD, AD & RD) by age groups for the 5 time points.

https://doi.org/10.1371/journal.pone.0247678.g003

diffusion of water molecules between myelinated axonal bundles. RD measures the diffusion perpendicular to the axons whereas AD measures the diffusion parallel to the axons. MD is an overall measure of the diffusion properties independent of the directions. The combination of the differences in FA (decrease) and MD (increase) is normally observed when there are increases in the extracellular space between the axons due to axonal degeneration or edema [13, 14]. Changes in AD and RD usually reflect specific directional changes in diffusivity parallel or perpendicular to the axons respectively. Our data showed a significant transient decrease in FA and increases in MD, AD and RD during sevoflurane anesthesia. These results suggest a widening of the inter-axonal space (Fig 4). These transient changes were highly significant and were detected diffusely throughout the brain white matter regions using both a voxel-wise

Table 2. Sex differences in changes in DTI metrics between baseline and 40 minutes after induction of anesthesia.

	Sex	N	Mean	Std Dev	Median	25th Pctl	75th Pctl
FA	F	30	-0.011	0.0044	-0.0108	-0.0133	-0.0083
	M	38	-0.010	0.0037	-0.0098	-0.0133	-0.0072
MD	F	30	0.000012	0.000008	0.000011	0.000007	0.000016
	M	38	0.000014	0.000008	0.000012	0.000009	0.000018
AD	F	30	0.000004	0.000011	0.000005	-0.000003	0.000011
	M	38	0.000008	0.000011	0.000006	0.000002	0.000013
RD	F	30	0.000015	0.000007	0.000015	0.000012	0.000019
	M	38	0.000017	0.000007	0.000015	0.000012	0.000021

https://doi.org/10.1371/journal.pone.0247678.t002

Table 3. Multiple linear regression was used to investigate the relationship between age and sex and the change in the DTI metrics (FA, MD, AD, RD), calculated as the value at 40 minutes after induction of anesthesia minus the baseline awake value.

	Parameter	Estimated β	Lower 95% CI	Upper 95% CI	P-Value
FA	Age	3.00E-06	-9.80E-05	1.04E-04	0.950
	Sex F	-1.78E-04	-2.18E-03	1.83E-03	0.860
	PRE	-1.868E-02	-7.87E-02	4.13E-02	0.536
MD	Age	-6.23E-08	-2.87E-07	1.62E-07	0.582
	Sex F	-2.56E-06	-6.56E-06	1.43E-06	0.205
	PRE	-1.79E-02	-7.65E-02	4.07E-02	0.544
AD	Age	2.50E-08	-2.66E-07	3.16E-07	0.864
	Sex F	-4.90E-06	-1.02E-05	4.42E-07	0.072
	PRE	-6.88E-02	-1.42E-01	4.27E-03	0.065
RD	Age	-6.23E-08	-2.87E-07	1.62E-07	0.582
	Sex F	-2.56E-06	-6.56E-06	1.43E-06	0.205
	PRE	-1.79E-02	-7.65E-02	4.07E-02	0.544

There were no significant differences with sex or age.

https://doi.org/10.1371/journal.pone.0247678.t003

technique (TBSS) (Fig 1) as well as regions of interest approaches (the Johns Hopkins Atlas ROIs) (Fig 2 and Tables 1 and 4). Our data also showed the well-known finding of declining FA and increases of MD with age after the 4th decade of life (Fig 3A) [13, 15–17]. Interestingly, the magnitude of the changes in FA, MD, AD and RD during anesthesia were the same for all age groups (Fig 3B, Tables 2 and 3), there were no age or sex associations with the effects of the anesthesia. Volumetric analysis suggests an overall shrinkage of white matter volumes and increases of gray matter volume (Table 5) while CSF volume showed a significant increase (Table 6) during anesthesia. This is the first time that these changes in white matter micro-structure have been reported during general anesthesia and the underlying physiology is not well-understood.

We review here several possible mechanisms consistent with the changes in the DTI metrics that we have detected:

Shrinking glial cells due to reduced activity

Previous studies exploring the sensitivity of DTI to functional activation have shown significant correlations between FA and task based activity (functional DTI or fDTI) in thalamocortical and optic tracts for tactile and visual stimuli respectively [18, 19]. Changes in glial cell morphology during activation may contribute to the physical changes of the extracellular space [20]. Unlike the BOLD effect in functional MRI [21] which is sensitive to blood oxygenation changes upon brain activation, DTI metrics such as FA, MD, RA and AD measure physical and geometrical attributes of the underlying tissue. The positive correlation of FA with brain activity [18] was believed to be due to glial cell swelling which changes the extra cellular space geometry. Shrinkage of the extracellular space upon neuronal firing has been studied for many years [22]. In particular, astrocytes, being the most abundant of glia cells in the brain, swell upon hyperactivity due to imbalances in Na+ and K+ concentrations inside and outside of the cells [23, 24]. In another study using diffusion weighted imaging on a rat optic nerve it was shown that levels of potassium concentrations in the extracellular space could control the diffusivity measures [25]. They also observed that the changes in diffusivity in the radial direction (similar to our RD measure) were larger than in the parallel direction (similar to our AD measure). In our study the changes in AD, although significant, the % change was much less

Table 4. Statistical comparisons of the 48 white matter ROIs by the Johns Hopkins Atlas for the different time point pairs.

	Pre-A1			Pre-A2			Pre-D2			Pre-D7			A1-A2		
**Region of Interest**	**tstat**	**p-value**	**diff**	**tstat**	**p-value**	**diff**	**tstat**	**p-value**	**diff**	**tstat**	**p-value**	**diff**	**tstat**	**p-value**	**diff**
Middle.cerebellar.peduncle	-9.1262	**2.2756E-13**	-0.0254	-8.6354	**1.7256E-12**	-0.0238	-1.9269	5.8233E-02	-0.0052	4.5911	**1.9956E-05**	0.0146	3.5393	**7.3457E-04**	0.0016
Pontine.crossing.tract..a.part.of.MCP.	12.6599	**1.9486E-19**	0.0343	10.4572	**1.0161E-15**	0.0308	-8.1996	**1.0507E-11**	-0.0259	1.9632	5.3778E-02	0.0069	-3.2719	**1.6905E-03**	-0.0035
Genu.of.corpus.callosum	17.7870	**4.4639E-27**	0.0104	21.3089	**1.4919E-31**	0.0123	0.0940	9.2542E-01	0.0001	0.6360	5.2694E-01	0.0007	5.4411	**8.0685E-07**	0.0018
Body.of.corpus.callosum	12.7469	**1.4063E-19**	0.0110	15.8670	**2.2095E-24**	0.0137	1.0212	3.1082E-01	0.0009	0.8763	3.8399E-01	0.0011	7.0589	**1.1884E-09**	0.0027
Splenium.of.corpus.callosum	17.0967	**3.9487E-26**	0.0126	19.4728	**2.7299E-29**	0.0150	-0.5829	5.6191E-01	-0.0005	0.5594	5.7773E-01	0.0009	5.8760	**1.4556E-07**	0.0024
Fornix..column.and.body.of.fornix.	7.8372	**4.7315E-11**	0.0116	9.2470	**1.3848E-13**	0.0133	2.5501	1.3064E-02	0.0034	2.3444	**2.2030E-02**	0.0040	1.7258	8.8995E-02	0.0016
Corticospinal.tract.R	13.8332	**2.5963E-21**	0.0426	16.3608	**4.2941E-25**	0.0478	-8.6303	**1.7626E-12**	-0.0334	-3.7032	**4.3277E-04**	-0.0166	2.5474	**1.3155E-02**	0.0051
Corticospinal.tract.L	15.9815	**1.5071E-24**	0.0440	18.9881	**1.1446E-28**	0.0515	-9.1014	**2.5198E-13**	-0.0322	-1.3301	1.8798E-01	-0.0078	4.6550	**1.5799E-05**	0.0074
Medial.lemniscus.R	19.0499	**9.5194E-29**	0.0683	17.6236	**7.4421E-27**	0.0659	-5.2352	**1.7879E-06**	-0.0155	-1.6530	1.0301E-01	-0.0124	-2.2362	**2.8670E-02**	-0.0024
Medial.lemniscus.L	20.9969	**3.5303E-31**	0.0718	20.1507	**3.8325E-30**	0.0699	-5.1350	**2.6237E-06**	-0.0191	-2.5014	**1.4821E-02**	-0.0212	-1.6779	9.8024E-02	-0.0019
Inferior.cerebellar.peduncle.R	-3.7803	**3.3590E-04**	-0.0133	-1.8634	6.6796E-02	-0.0065	-6.0035	**8.7465E-08**	-0.0214	2.6971	**8.8395E-03**	0.0121	5.0176	**4.0964E-06**	0.0068
Inferior.cerebellar.peduncle.L	-0.7856	4.3488E-01	-0.0030	0.2453	8.0696E-01	0.0010	1.2767	**2.0611E-01**	0.0032	18.0103	**2.2311E-27**	0.0773	2.5496	**1.3080E-02**	0.0040
Superior.cerebellar.peduncle.R	11.5231	**1.5057E-17**	0.0256	12.0462	**1.9976E-18**	0.0280	7.7841	**5.8978E-11**	0.0143	-0.1618	8.7194E-01	-0.0009	2.8598	**5.6466E-03**	0.0024
Superior.cerebellar.peduncle.L	13.6635	**4.7967E-21**	0.0300	14.6504	**1.4238E-22**	0.0322	14.6385	**1.4840E-22**	0.0232	-4.5292	**2.4985E-05**	-0.0225	2.5270	**1.3871E-02**	0.0022
Cerebral.peduncle.R	11.0236	**1.0672E-16**	0.0121	11.7606	**5.9940E-18**	0.0130	0.1044	9.1713E-01	0.0001	1.2102	2.3044E-01	0.0033	1.5221	1.3268E-01	0.0009
Cerebral.peduncle.L	10.8710	**1.9523E-16**	0.0111	11.6737	**8.3912E-18**	0.0117	0.7856	4.3488E-01	0.0009	0.7420	4.6068E-01	0.0028	0.8793	3.8236E-01	0.0005
Anterior.limb.of.internal.capsule.R	12.9157	**7.4885E-20**	0.0108	15.2070	**2.0683E-23**	0.0129	0.0139	9.8899E-01	0.0000	0.6467	5.2002E-01	0.0008	5.2958	**1.4162E-06**	0.0020
Anterior.limb.of.internal.capsule.L	14.3838	**3.6361E-22**	0.0106	14.9851	**4.4420E-23**	0.0117	1.1154	2.6867E-01	0.0009	1.1441	2.5664E-01	0.0013	2.7409	**7.8484E-03**	0.0011
Posterior.limb.of.internal.capsule.R	16.6291	**1.7851E-25**	0.0166	17.6387	**7.0962E-27**	0.0183	0.6917	4.9154E-01	0.0007	1.1328	2.6134E-01	0.0018	4.1879	**8.4044E-05**	0.0016
Posterior.limb.of.internal.capsule.L	16.1854	**7.6569E-25**	0.0123	17.7376	**5.2084E-27**	0.0140	0.0262	9.7919E-01	0.0000	0.3456	7.3075E-01	0.0005	4.1415	**9.8774E-05**	0.0017
Retrolenticular.part.of.internal.capsule.R	16.3333	**4.7003E-25**	0.0179	16.7208	**1.3255E-25**	0.0193	1.3283	1.8859E-01	0.0018	0.1624	8.7151E-01	0.0003	2.9207	**4.7542E-03**	0.0014
Retrolenticular.part.of.internal.capsule.L	7.7812	**5.9700E-11**	0.0121	8.6751	**1.4643E-12**	0.0130	-0.3730	7.1029E-01	-0.0004	0.2945	7.6932E-01	0.0007	1.5365	1.2911E-01	0.0009
Anterior.corona.radiata.R	15.2749	**1.6392E-23**	0.0090	15.0101	**4.0749E-23**	0.0100	1.0346	3.0460E-01	0.0008	0.9016	3.7048E-01	0.0009	3.1669	**2.3199E-03**	0.0011
Anterior.corona.radiata.L	11.9238	**3.1954E-18**	0.0071	11.9041	**3.4473E-18**	0.0079	0.6193	5.3781E-01	0.0004	0.9847	3.2829E-01	0.0011	2.6944	**8.9046E-03**	0.0008
Superior.corona.radiata.R	16.8404	**8.9987E-26**	0.0114	18.0527	**1.9572E-27**	0.0124	0.3992	6.9102E-01	0.0003	0.7596	4.5014E-01	0.0008	3.1914	**2.1558E-03**	0.0011
Superior.corona.radiata.L	17.6042	**7.9083E-27**	0.0108	18.9524	**1.2733E-28**	0.0124	0.6374	5.2602E-01	0.0004	0.5880	5.5849E-01	0.0006	5.1001	**2.9963E-06**	0.0015
Posterior.corona.radiata.R	16.5913	**2.0189E-25**	0.0117	19.5151	**2.4115E-29**	0.0133	1.2844	2.0342E-01	0.0010	0.9371	3.5205E-01	0.0012	4.2280	**7.3048E-05**	0.0016
Posterior.corona.radiata.L	17.6404	**7.0593E-27**	0.0102	18.3170	**8.6882E-28**	0.0114	0.2206	8.2609E-01	0.0001	0.7944	4.2977E-01	0.0010	3.6439	**5.2492E-04**	0.0012
Posterior.thalamic.radiation.R	15.7533	**3.2359E-24**	0.0122	18.0177	**2.1811E-27**	0.0140	1.5005	1.3819E-01	0.0014	0.2541	8.0017E-01	0.0004	5.0556	**3.5486E-06**	0.0017
Posterior.thalamic.radiation.L	11.8964	**3.5500E-18**	0.0099	15.2176	**1.9948E-23**	0.0121	-0.0493	9.6079E-01	0.0000	1.1020	2.7442E-01	0.0013	6.4184	**1.6396E-08**	0.0021
Sagittal.stratum.R	11.2313	**4.7109E-17**	0.0119	12.5739	**2.6923E-19**	0.0139	1.6721	9.9176E-02	0.0018	0.7756	4.4069E-01	0.0016	3.2109	**2.0333E-03**	0.0020

(*Continued*)

Table 4. (Continued)

	Pre-A1			Pre-A2			Pre-D2			Pre-D7			A1-A2		
**Region of Interest**	**tstat**	**p-value**	**diff**	**tstat**	**p-value**	**diff**	**tstat**	**p-value**	**diff**	**tstat**	**p-value**	**diff**	**tstat**	**p-value**	**diff**
Sagittal.stratum.L	10.2353	**2.4787E-15**	0.0081	11.0947	**8.0635E-17**	0.0089	1.2304	2.2285E-01	0.0012	1.5945	1.1554E-01	0.0032	1.4310	1.5707E-01	0.0007
External.capsule.R	14.4599	**2.7800E-22**	0.0092	12.9036	**7.8343E-20**	0.0096	0.9711	3.3498E-01	0.0009	1.2101	2.3048E-01	0.0013	1.3466	1.8265E-01	0.0004
External.capsule.L	11.7365	**6.5787E-18**	0.0080	12.5250	**3.2373E-19**	0.0079	1.0025	3.1971E-01	0.0006	0.9206	3.6058E-01	0.0013	-0.3574	7.2195E-01	-0.0001
Cingulum..cingulate. gyrus..R	9.9238	**8.7333E-15**	0.0091	10.8180	**2.4090E-16**	0.0110	1.9750	5.2395E-02	0.0018	0.7865	4.3434E-01	0.0010	3.0003	**3.7849E-03**	0.0019
Cingulum..cingulate. gyrus..L	10.1565	**3.4056E-15**	0.0081	11.5322	**1.4532E-17**	0.0095	-0.8958	3.7356E-01	-0.0007	0.3275	7.4430E-01	0.0005	2.7051	**8.6496E-03**	0.0013
Cingulum.. hippocampus..R	8.0776	**1.7435E-11**	0.0093	8.1270	**1.4202E-11**	0.0108	2.3744	**2.0447E-02**	0.0030	1.9577	5.4438E-02	0.0045	1.6322	**1.0734E-01**	0.0014
Cingulum.. hippocampus..L	4.5659	**2.1869E-05**	0.0060	6.9327	**1.9985E-09**	0.0084	0.5085	6.1277E-01	0.0007	0.9029	3.6983E-01	0.0024	3.7963	**3.1858E-04**	0.0024
Fornix..cres. . . .Stria. terminalis.R	8.7587	**1.0362E-12**	0.0106	9.9927	**6.6056E-15**	0.0129	1.4098	1.6322E-01	0.0018	1.7206	8.9935E-02	0.0032	3.1427	**2.4934E-03**	0.0023
Fornix..cres. . . .Stria. terminalis.L	6.0362	**7.6740E-08**	0.0088	6.6659	**5.9734E-09**	0.0099	1.3253	1.8957E-01	0.0023	0.9384	3.5142E-01	0.0023	1.4670	1.4704E-01	0.0011
Superior.longitudinal. fasciculus.R	18.3319	**8.3032E-28**	0.0115	19.0502	**9.5112E-29**	0.0122	1.6419	1.0529E-01	0.0011	0.8852	3.7920E-01	0.0010	1.9507	5.5277E-02	0.0007
Superior.longitudinal. fasciculus.L	12.2758	**8.3158E-19**	0.0087	16.2023	**7.2406E-25**	0.0103	-0.7031	4.8442E-01	-0.0004	0.2406	8.1060E-01	0.0003	4.2880	**5.9167E-05**	0.0016
Superior.fronto. occipital.fasciculus.R	8.1819	**1.1310E-11**	0.0082	9.4445	**6.1617E-14**	0.0100	-1.3281	1.8865E-01	-0.0013	-1.0899	2.7964E-01	-0.0013	2.4779	**1.5741E-02**	0.0018
Superior.fronto. occipital.fasciculus.L	4.7591	**1.0770E-05**	0.0060	6.0560	**7.0888E-08**	0.0079	-0.5052	6.1506E-01	-0.0006	-0.1136	9.0990E-01	-0.0002	2.0901	**4.0405E-02**	0.0019
Uncinate.fasciculus.R	5.7290	**2.6077E-07**	0.0081	6.2181	**3.6914E-08**	0.0085	-0.2483	8.0469E-01	-0.0003	0.8655	3.8987E-01	0.0023	0.4105	6.8276E-01	0.0004
Uncinate.fasciculus.L	5.6344	**3.7876E-07**	0.0072	6.0927	**6.1155E-08**	0.0077	0.2388	8.1196E-01	0.0003	0.8173	4.1667E-01	0.0021	0.5464	5.8660E-01	0.0005
Tapetum.R	11.3724	**2.7106E-17**	0.0103	12.3854	**5.4867E-19**	0.0108	1.1157	2.6854E-01	0.0008	1.1200	2.6673E-01	0.0016	0.8858	3.7889E-01	0.0005
Tapetum.L	6.4521	**1.4297E-08**	0.0060	9.4271	**6.6155E-14**	0.0084	-0.0606	9.5183E-01	-0.0001	1.4514	1.5134E-01	0.0013	2.9667	**4.1699E-03**	0.0025

Pre: Before anesthesia, A1: 40 mins after administration of anesthesia, A2: 100 mins after administration of anesthesia, D2: 1 day after anesthesia, D7: 7 days after anesthesia. Significant differences highlighted in bold.

https://doi.org/10.1371/journal.pone.0247678.t004

than MD or RD (Table 1). Increases in extracellular potassium concentrations with neuronal activity have been well described before [26, 27]. The earlier fDTI study showed an increase in FA upon neuronal activation; our study showed decreased FA under sevoflurane anesthesia. One possibility is that the reduced brain activity in the anesthesia state causes potassium concentration levels to drop below baseline and thereby causes glial cells to shrink. Indeed, as we will discuss in the next section on the glymphatic system, glial cells shrink during sleep to make room for clearance of waste material in the brain [28]. This shrinkage would lead increased extracellular space, and MD, RD and AD would increase and FA would decrease. The volumetric results (Table 6) that showed increased CSF volume and an overall trend in increased ventricular size may corroborate this interpretation. In addition, we showed that the volumes of several white matter regions were reduced during anesthesia. This reduction might be related to the shrinkage of the glial cells. It should be noted that DTI metrics such as FA that measure the microstructure of white matter does not have a direct relationship with white matter volumetric measures; the increase in extracellular space does not necessarily contradict the reduction of overall white matter volume [29, 30].

The glymphatic system

The glymphatic system is involved with clearance of waste matter in the central nervous system [31–33]. It is part of the perivascular system that drives CSF into brain parenchyma and

Table 5. Volumetric comparisons between day 7 and during anesthesia (A2).

White Matter Regions	tstat	p-value	diff	%change	Gray Matter Regions	tstat	p-value	diff	%change
wm.lh.bankssts	0.081	9.354E-01	2.162	-0.073	bankssts	5.353	**1.140E-06**	80.088	-3.285
wm.lh.caudalanteriorcingulate	-0.297	7.676E-01	-8.876	0.345	caudalanteriorcingulate	0.222	8.247E-01	3.706	-0.228
wm.lh.caudalmiddlefrontal	-3.229	**1.926E-03**	-205.525	3.204	caudalmiddlefrontal	0.235	8.149E-01	17.868	-0.302
wm.lh.cuneus	-3.091	**2.909E-03**	-68.284	2.620	cuneus	1.447	1.525E-01	30.941	-0.975
wm.lh.entorhinal	2.168	**3.375E-02**	30.219	-3.479	entorhinal	2.609	**1.120E-02**	74.794	-3.742
wm.lh.fusiform	0.302	7.635E-01	12.413	-0.198	fusiform	5.594	**4.430E-07**	333.029	-3.422
wm.lh.inferiorparietal	-1.668	9.998E-02	-123.734	1.266	inferiorparietal	6.209	**3.830E-08**	388.603	-3.160
wm.lh.inferiortemporal	0.017	9.867E-01	0.634	-0.010	inferiortemporal	5.264	**1.600E-06**	387.750	-3.759
wm.lh.isthmuscingulate	0.007	9.948E-01	0.176	-0.005	isthmuscingulate	1.233	2.218E-01	22.279	-0.864
wm.lh.lateraloccipital	-3.379	**1.217E-03**	-376.244	3.707	lateraloccipital	3.935	**2.000E-04**	246.853	-2.014
wm.lh.lateralorbitofrontal	0.689	4.935E-01	31.135	-0.468	lateralorbitofrontal	6.832	**3.020E-09**	248.868	-3.289
wm.lh.lingual	-1.544	1.273E-01	-59.632	1.117	lingual	6.553	**9.470E-09**	193.559	-2.875
wm.lh.medialorbitofrontal	-0.439	6.618E-01	-18.801	0.497	medialorbitofrontal	1.497	1.390E-01	55.824	-1.084
wm.lh.middletemporal	1.433	1.564E-01	54.701	-1.006	middletemporal	6.053	**7.170E-08**	502.132	-4.765
wm.lh.parahippocampal	1.162	2.494E-01	11.321	-0.757	parahippocampal	4.734	**1.180E-05**	81.603	-4.056
wm.lh.paracentral	-1.810	7.480E-02	-65.051	1.645	paracentral	0.847	3.999E-01	26.147	-0.739
wm.lh.parsopercularis	-2.525	**1.394E-02**	-63.713	1.857	parsopercularis	2.346	**2.196E-02**	52.529	-1.156
wm.lh.parsorbitalis	-1.481	1.433E-01	-17.015	1.626	parsorbitalis	3.944	**1.941E-04**	77.912	-3.363
wm.lh.parstriangularis	-3.147	**2.465E-03**	-60.168	2.008	parstriangularis	3.694	**4.458E-04**	70.279	-1.989
wm.lh.pericalcarine	-3.140	**2.516E-03**	-76.857	2.507	pericalcarine	0.617	5.391E-01	12.647	-0.590
wm.lh.postcentral	-1.932	5.763E-02	-111.412	1.452	postcentral	4.601	**1.920E-05**	233.324	-2.428
wm.lh.posteriorcingulate	-0.907	3.674E-01	-29.943	0.678	posteriorcingulate	0.324	7.473E-01	6.809	-0.227
wm.lh.precentral	-4.929	**5.710E-06**	-567.278	4.094	precentral	4.610	**1.860E-05**	513.647	-3.716
wm.lh.precuneus	-1.333	1.871E-01	-91.800	1.039	precuneus	3.868	**2.508E-04**	214.221	-2.216
wm.lh.rostralanteriorcingulate	1.392	1.684E-01	37.394	-1.545	rostralanteriorcingulate	1.140	2.585E-01	35.735	-1.442
wm.lh.rostralmiddlefrontal	-4.133	**1.016E-04**	-420.491	3.423	rostralmiddlefrontal	1.633	1.072E-01	116.338	-0.803
wm.lh.superiorfrontal	-3.255	**1.779E-03**	-537.750	3.103	superiorfrontal	-0.158	8.751E-01	-22.956	0.108
wm.lh.superiorparietal	-2.265	**2.672E-02**	-273.254	2.233	superiorparietal	3.886	**2.359E-04**	350.632	-2.641
wm.lh.superiortemporal	-1.089	2.799E-01	-65.050	0.783	superiortemporal	6.719	**4.800E-09**	443.118	-3.467
wm.lh.supramarginal	-1.082	2.832E-01	-88.826	0.980	supramarginal	4.124	**1.051E-04**	303.779	-2.710
wm.lh.frontalpole	-2.010	**4.843E-02**	-8.896	3.347	frontalpole	-2.789	**6.882E-03**	-40.941	4.227
wm.lh.temporalpole	-1.542	1.278E-01	-15.319	2.313	temporalpole	1.850	6.868E-02	83.088	-3.455
wm.lh.transversetemporal	-0.549	5.847E-01	-8.178	0.951	transversetemporal	4.309	**5.480E-05**	44.706	-3.843
wm.lh.insula	-0.752	4.549E-01	-37.959	0.389	insula	2.243	**2.819E-02**	75.294	-1.072
wm.rh.bankssts	0.595	5.537E-01	12.163	-0.435	bankssts	4.625	**1.760E-05**	89.559	-4.034
wm.rh.caudalanteriorcingulate	0.343	7.324E-01	9.994	-0.390	caudalanteriorcingulate	-0.790	4.324E-01	-12.779	0.712
wm.rh.caudalmiddlefrontal	-1.490	1.409E-01	-99.388	1.681	caudalmiddlefrontal	2.202	**3.113E-02**	158.529	-2.656
wm.rh.cuneus	-4.176	**8.770E-05**	-79.881	2.931	cuneus	-0.080	9.363E-01	-2.059	0.059
wm.rh.entorhinal	2.358	**2.132E-02**	23.166	-2.904	entorhinal	2.408	**1.881E-02**	70.691	-3.646
wm.rh.fusiform	0.549	5.849E-01	22.657	-0.367	fusiform	6.037	**7.640E-08**	356.103	-3.735
wm.rh.inferiorparietal	-3.354	**1.316E-03**	-304.735	2.656	inferiorparietal	7.046	**1.250E-09**	515.868	-3.409
wm.rh.inferiortemporal	-0.215	8.307E-01	-7.449	0.126	inferiortemporal	5.182	**2.190E-06**	298.985	-2.946
wm.rh.isthmuscingulate	-1.842	6.988E-02	-50.916	1.496	isthmuscingulate	0.386	7.009E-01	6.926	-0.287
wm.rh.lateraloccipital	-4.164	**9.120E-05**	-329.579	3.228	lateraloccipital	4.216	**7.630E-05**	295.338	-2.379
wm.rh.lateralorbitofrontal	1.139	2.588E-01	95.641	-1.362	lateralorbitofrontal	5.302	**1.380E-06**	256.074	-3.413
wm.rh.lingual	-2.246	**2.804E-02**	-87.431	1.531	lingual	2.640	**1.030E-02**	91.015	-1.259
wm.rh.medialorbitofrontal	1.042	3.009E-01	36.841	-0.988	medialorbitofrontal	1.402	1.656E-01	52.471	-0.972

(*Continued*)

Table 5. (Continued)

White Matter Regions	tstat	p-value	diff	%change	Gray Matter Regions	tstat	p-value	diff	%change
wm.rh.middletemporal	0.076	9.400E-01	3.079	-0.049	middletemporal	7.015	**1.430E-09**	413.824	-3.591
wm.rh.parahippocampal	1.762	8.261E-02	21.209	-1.379	parahippocampal	4.931	**5.670E-06**	80.309	-4.123
wm.rh.paracentral	-1.852	6.839E-02	-111.200	2.367	paracentral	1.798	7.667E-02	47.471	-1.205
wm.rh.parsopercularis	-1.137	2.597E-01	-42.666	1.307	parsopercularis	2.917	**4.803E-03**	101.647	-2.582
wm.rh.parsorbitalis	-1.211	2.302E-01	-21.096	1.648	parsorbitalis	5.471	**7.190E-07**	94.162	-3.487
wm.rh.parstriangularis	-4.285	**5.990E-05**	-90.421	2.656	parstriangularis	2.822	**6.277E-03**	60.132	-1.420
wm.rh.pericalcarine	-2.016	**4.783E-02**	-60.822	1.822	pericalcarine	2.142	**3.580E-02**	45.574	-1.833
wm.rh.postcentral	-2.236	**2.869E-02**	-144.635	1.917	postcentral	4.355	**4.670E-05**	190.074	-2.052
wm.rh.posteriorcingulate	-1.959	5.425E-02	-57.569	1.389	posteriorcingulate	0.214	8.311E-01	4.985	-0.163
wm.rh.precentral	-4.330	**5.110E-05**	-537.878	3.858	precentral	4.649	**1.620E-05**	494.897	-3.681
wm.rh.precuneus	-4.148	**9.650E-05**	-249.516	2.693	precuneus	1.902	6.142E-02	139.382	-1.387
wm.rh.rostralanteriorcingulate	0.599	5.514E-01	9.610	-0.517	rostralanteriorcingulate	1.222	2.260E-01	25.868	-1.432
wm.rh.rostralmiddlefrontal	-1.814	7.420E-02	-265.241	2.032	rostralmiddlefrontal	1.696	9.458E-02	205.824	-1.370
wm.rh.superiorfrontal	-1.288	2.022E-01	-207.425	1.220	superiorfrontal	1.614	1.112E-01	233.765	-1.137
wm.rh.superiorparietal	-4.960	**5.100E-06**	-470.591	3.948	superiorparietal	2.396	1.935E-02	227.412	-1.728
wm.rh.superiortemporal	-1.682	9.724E-02	-105.721	1.515	superiortemporal	7.441	**2.450E-10**	316.191	-2.704
wm.rh.supramarginal	-2.688	**9.070E-03**	-199.659	2.299	supramarginal	4.199	**8.070E-05**	339.559	-3.307
wm.rh.frontalpole	-3.039	**3.385E-03**	-18.993	5.704	frontalpole	-2.823	**6.260E-03**	-48.382	4.188
wm.rh.temporalpole	0.971	3.352E-01	9.363	-1.364	temporalpole	1.611	1.119E-01	57.456	-2.262
wm.rh.transversetemporal	0.288	7.740E-01	2.841	-0.433	transversetemporal	3.647	**5.200E-04**	33.235	-3.576
wm.rh.insula	-0.743	4.602E-01	-63.557	0.661	insula	1.285	2.032E-01	78.059	-1.110
Left.UnsegmentedWhiteMatter	-1.546	1.269E-01	-269.732	0.976					
Right.UnsegmentedWhiteMatter	-1.023	3.099E-01	-179.632	0.640					
Left.hemisphere.cerebral.white.matter.Vol	-2.420	**1.825E-02**	-2982.322	1.349					
Right.hemisphere.cerebral.white.matter.Vol	-2.736	7.950E-03	-3230.282	1.454					
Estimated.Total.Intracranial.Vol	1.933	5.746E-02	10491.750	-0.639					

Regions of interests were computed using Freesurfer and a paired t-test was computed between the two time-points. Differences represent anesthesia (A2)-Day 7.

https://doi.org/10.1371/journal.pone.0247678.t005

drains waste solutes from the interstitial space fluid (ISF) into the perivascular space around the veins. Astrocytic endfeet cover almost the entire brain vasculature regulating endothelial tight junctions that form the blood brain barrier as well vascular tone through vasoactive agents [34]. Astrocytic endfeet also express water channels called aquaporin-4 (AQP4); these

Table 6. Comparisons of ventricular size during anesthesia (A2) and day 7.

Regions	tstat	p-value	diff	%change
Left.Lateral.Ventricle	0.720	0.474	44.768	-0.304
Left.Inf.Lat.Vent	1.352	0.181	14.551	-2.843
3rd.Ventricle	2.237	**0.029**	18.806	-1.310
4th.Ventricle	-3.746	**0.000**	-58.512	3.000
CSF	3.338	**0.001**	60.884	-4.893
Right.Lateral.Ventricle	1.137	0.260	61.112	-0.458
Right.Inf.Lat.Vent	1.624	0.109	9.740	-2.234
5th.Ventricle	-0.141	0.888	-0.013	18.367

Differences are Anesthesia (A2)—Day 7. Significant differences highlighted in bold.

https://doi.org/10.1371/journal.pone.0247678.t006

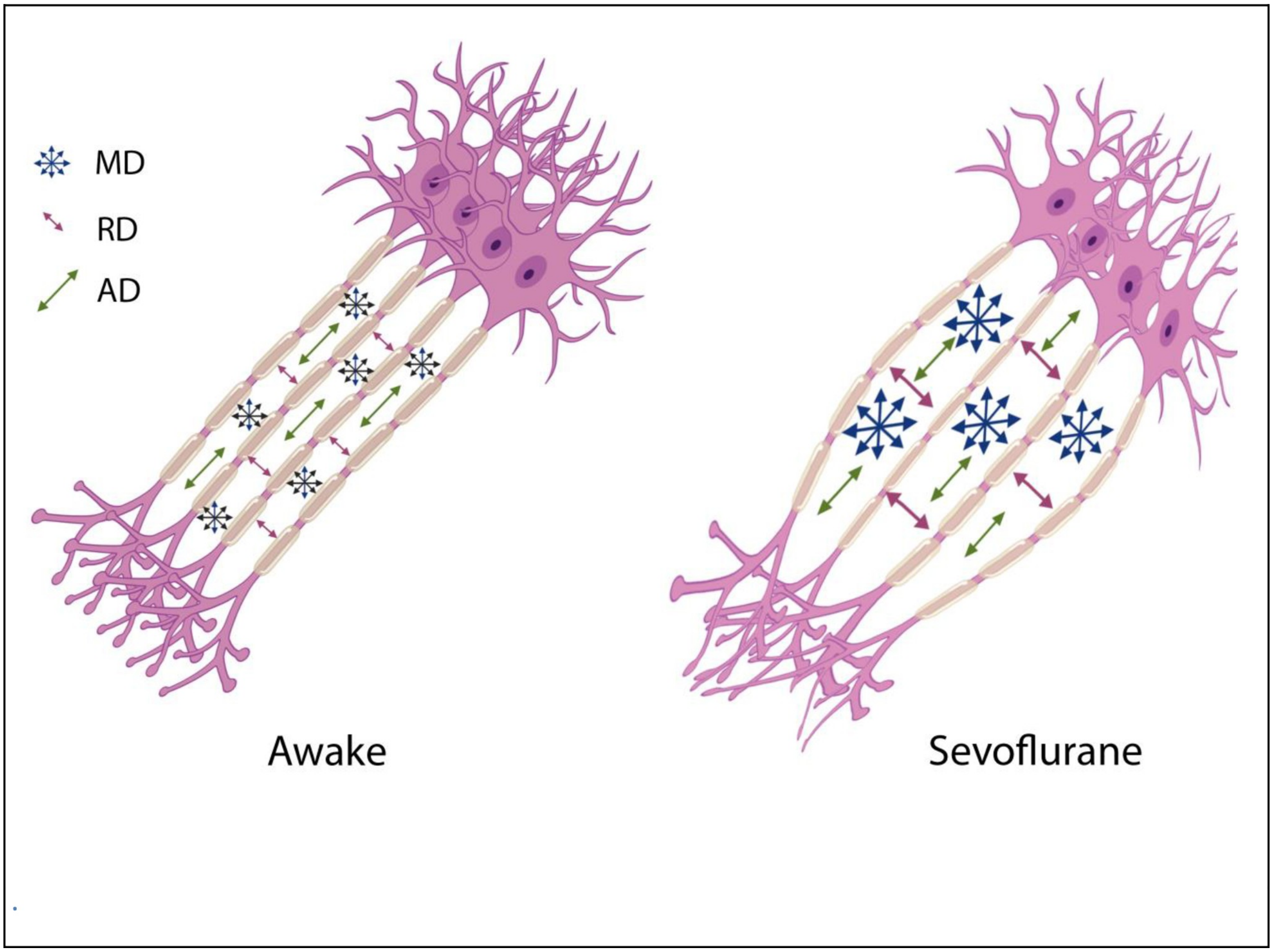

Fig 4. Illustration of one possible geometric implication of the DTI metrics. Increases in the interaxonal space will result in increased MD and RD, but to a lesser extend the AD.

https://doi.org/10.1371/journal.pone.0247678.g004

channels facilitate the movement of interstitial fluids into a perivascular space around draining veins [35]. AQP4 channels are mainly engaged during sleep and under anesthesia [28]. In addition, interstitial space was dilated during sleep and anesthesia states when compared to awake states [28]. Sevoflurane upregulates AQP4 expression [36]. It should be noted that different types of anesthesia seem to have different effects on the glymphatic transport system; dexmedetomidine (an alpha-2 adrenergic agonist) had 32% more enhanced glymphatic transport when compared with isoflurane [37]. In this study it was speculated that the lowered adrenergic tone with dexmedetomidine increased the interstitial fluid volume fraction which then facilitated glymphatic transport [38]. Using two-photon microscopy and fluorescent tracers, it was shown that the interstitial space was increased by 60% during sleep as well as under anesthesia using ketamine/xylazine in a mouse model [28]. We speculate that this increase in the ISF is the cause of the changes in the DTI metrics such as FA, MD, AD and RD. Increases in ISF would push axonal bundles apart and thereby reduce FA (a measure of bundle coherence), increase MD (increase of isotropic free water movement), and increase RA (the space perpendicular to the axons) [Fig 4]. Although AD was decreased but its change was only 0.4% vs 2.4% for RD. From a geometric point of view, increases in ISF has less effect on AD then RD [Fig 4]. In another study of normal pressure hydrocephalus (NPH), a lower FA was associated

with better glymphatic clearance [39]. In summary the increases of ISF is tightly coupled with the glial cell shrinkage suggested in the previous paragraph as it is believed that it is the response of astrocytes to a reduced wakefulness that is changing the ISF space [40].

The microtubule system

Microtubules are the major components of the cytoskeleton of most eukaryotic cells and in particular axonal structures [41, 42]. Several studies have shown the effects of diverse anesthetics on microtubule structural stability [43]. Among the mechanisms that have been proposed are Tau hyperphosphorylation [44–47]. Temporary impairment of Tau function by hyperphosphorylation at several positions can be induced by the administration of anesthetics. In one study, 2.5% sevoflurane in 5–6 month-old C57B16/J mice increased Tau phosphorylation level on Ser 396/404 at 1 h following anesthesia. This short-term increase in Tau phosphorylation was reversible as no significant increase was detected 1 day after anesthesia [44]. Isoflurane anesthesia in mice with tauopathy elevated phospho-tau for at least 1 week after the anesthesia [48]. A study on the effects of sevoflurane on young mice comparing wild type and Tau-KO showed that sevoflurane increased activation of glycogen synthase kinase 3β (the kinase that is related to Tau phosphorylation) in young WT resulting in cognitive impairment but not in the Tau-KO [46]. Another study showed the same anesthesia induced microtubule instability through the same GSK3β pathway using sevoflurane, urethane and ketamine [43]. The instability of microtubules is rather complex and it would be difficult to propose a geometric explanation for the changes that we have found in the different DTI metrics, but anesthesia induced microtubule instability could lead to deformation of axonal formations and affect DTI metrics that depend on structural coherence of axonal bundles.

Intracranial pressure

Another potential source of these changes might be vasodilatory effects of sevoflurane that give rise to microscopy vasogenic swelling and subsequent increase in water content of the extra-axonal space. Halothane, fentanyl, and thiopental can all cause an increase in brain water and electrolyte distribution [49, 50]. Sevoflurane is one of the more common inhaled anesthetics and like most other volatile anesthetics is known to cause vasodilation [51, 52]. Changes in intracranial pressure (ICP) following sevoflurane have also been reported but the results have been inconclusive [53–55]. Increases in ICP might be due to a combination of vasodilation and accumulation of CSF due to reduced reuptake [56]. It has been previously reported that the apparent diffusion coefficient (ADC) in deep white matter increases in idiopathic intracranial hypertension [57]. Diffusion imaging in hydrocephalus has shown increased diffusion coefficients in periventricular white matter [58, 59]. Increases in ADC reflect increases in extracellular water or increased fraction of mobile water in intracellular space. Increased ICP can cause increases in CSF in the extracellular space of white matter. This transepedymal pathway of CSF has been studied recently [60]. Although increased CSF in the extracellular space could explain some of our findings, targeted research in the relationship between ICP and FA have not provided any consistent results, some have shown a decrease [61, 62], increased [63] and some no change [64], so we tend to discount this explanation of our findings.

Conclusion

Although these results show that the changes in the white matter microstructure are transient, it is important to consider these results in research studies that necessitate the use of anesthesia, given that general anesthesia can produce significant changes in diffusion measures of white matter integrity as well as in volume of specific brain regions. We have suggested several

possible mechanisms for the observed changes in white matter microstructure as detected using standard in-vivo DTI techniques. To better unravel the underlying physiological changes more research need to be performed in vitro using imaging technologies such as electron microscopy (EM). In addition, investigations into whether these changes in white matter microstructure are related to loss of consciousness are warranted.

Acknowledgments

We thank Helen Chang, M.S., Senior Statistician, Icahn School of Medicine at Mount Sinai for her advice on some of the statistical analysis.

Author Contributions

Conceptualization: Min Yin Lun, Mary Sano, Stacie G. Deiner, Mark G. Baxter.

Data curation: Cheuk Y. Tang, Joshua S. Mincer, Johnny C. Ng, Jess W. Brallier, Arthur E. Schwartz, Helen Ahn, Patrick J. McCormick, Tommer Nir, Bradley Delman, Mary Sano, Stacie G. Deiner, Mark G. Baxter.

Formal analysis: Cheuk Y. Tang, Min Yin Lun, Johnny C. Ng.

Funding acquisition: Stacie G. Deiner, Mark G. Baxter.

Investigation: Mary Sano, Mark G. Baxter.

Methodology: Mark G. Baxter.

Project administration: Joshua S. Mincer, Mark G. Baxter.

Resources: Cheuk Y. Tang, Min Yin Lun, Joshua S. Mincer, Johnny C. Ng, Jess W. Brallier, Arthur E. Schwartz, Helen Ahn, Patrick J. McCormick, Tommer Nir, Bradley Delman, Mary Sano, Stacie G. Deiner.

Software: Cheuk Y. Tang.

Supervision: Joshua S. Mincer, Mark G. Baxter.

Writing – original draft: Cheuk Y. Tang.

Writing – review & editing: Cheuk Y. Tang, Min Yin Lun, Mark G. Baxter.

References

1. Jesse S, Muller HP, Schoen M, Asoglu H, Bockmann J, Huppertz HJ, Rasche V, Ludolph AC, Boeckers TM, Kassubek J: Severe white matter damage in SHANK3 deficiency: a human and translational study. *Ann Clin Transl Neurol* 2020, 7(1):46–58. https://doi.org/10.1002/acn3.50959 PMID: 31788990
2. Artuso R, Mencarelli MA, Polli R, Sartori S, Ariani F, Pollazzon M, Marozza A, Cilio MR, Specchio N, Vigevano F et al: Early-onset seizure variant of Rett syndrome: definition of the clinical diagnostic criteria. *Brain Dev* 2010, 32(1):17–24. https://doi.org/10.1016/j.braindev.2009.02.004 PMID: 19362436
3. Veenith T, Coles JP: Anaesthesia for magnetic resonance imaging and positron emission tomography. *Curr Opin Anaesthesiol* 2011, 24(4):451–458. https://doi.org/10.1097/ACO.0b013e328347e373 PMID: 21577098
4. Schroeck H, Welch TL, Rovner MS, Johnson HA, Schroeck FR: Anesthetic challenges and outcomes for procedures in the intraoperative magnetic resonance imaging suite: A systematic review. *J Clin Anesth* 2019, 54:89–101. https://doi.org/10.1016/j.jclinane.2018.10.022 PMID: 30415150
5. Mincer JS, Baxter MG, McCormick PJ, Sano M, Schwartz AE, Brallier JW, Allore HG, Delman BN, Sewell MC, Kundu P et al: Delineating the Trajectory of Cognitive Recovery From General Anesthesia in Older Adults: Design and Rationale of the TORIE (Trajectory of Recovery in the Elderly) Project. *Anesth Analg* 2018, 126(5):1675–1683. https://doi.org/10.1213/ANE.0000000000002427 PMID: 28891911

6. Newman S, Stygall J, Hirani S, Shaefi S, Maze M: Postoperative cognitive dysfunction after noncardiac surgery: a systematic review. *Anesthesiology* 2007, 106(3):572–590. https://doi.org/10.1097/00000542-200703000-00023 PMID: 17325517

7. Mason SE, Noel-Storr A, Ritchie CW: The impact of general and regional anesthesia on the incidence of post-operative cognitive dysfunction and post-operative delirium: a systematic review with meta-analysis. *J Alzheimers Dis* 2010, 22 Suppl 3:67–79. https://doi.org/10.3233/JAD-2010-101086 PMID: 20858956

8. Smith SM, Jenkinson M, Johansen-Berg H, Rueckert D, Nichols TE, Mackay CE, Watkins KE, Ciccarelli O, Cader MZ, Matthews PM et al: Tract-based spatial statistics: voxelwise analysis of multi-subject diffusion data. *Neuroimage* 2006, 31(4):1487–1505. https://doi.org/10.1016/j.neuroimage.2006.02.024 PMID: 16624579

9. Smith SM, Nichols TE: Threshold-free cluster enhancement: addressing problems of smoothing, threshold dependence and localisation in cluster inference. *Neuroimage* 2009, 44(1):83–98. https://doi.org/10.1016/j.neuroimage.2008.03.061 PMID: 18501637

10. Desikan RS, Segonne F, Fischl B, Quinn BT, Dickerson BC, Blacker D, Buckner RL, Dale AM, Maguire RP, Hyman BT et al: An automated labeling system for subdividing the human cerebral cortex on MRI scans into gyral based regions of interest. *Neuroimage* 2006, 31(3):968–980. https://doi.org/10.1016/j.neuroimage.2006.01.021 PMID: 16530430

11. Peled S: New perspectives on the sources of white matter DTI signal. *IEEE Trans Med Imaging* 2007, 26(11):1448–1455. https://doi.org/10.1109/TMI.2007.906787 PMID: 18041260

12. Clark CA, Le Bihan D: Water diffusion compartmentation and anisotropy at high b values in the human brain. *Magn Reson Med* 2000, 44(6):852–859. https://doi.org/10.1002/1522-2594(200012)44:6<852::aid-mrm5>3.0.co;2-a PMID: 11108621

13. Bennett IJ, Madden DJ, Vaidya CJ, Howard DV, Howard JH Jr.: Age-related differences in multiple measures of white matter integrity: A diffusion tensor imaging study of healthy aging. *Hum Brain Mapp* 2010, 31(3):378–390. https://doi.org/10.1002/hbm.20872 PMID: 19662658

14. Filippi M, Iannucci G, Cercignani M, Assunta Rocca M, Pratesi A, Comi G: A quantitative study of water diffusion in multiple sclerosis lesions and normal-appearing white matter using echo-planar imaging. *Arch Neurol* 2000, 57(7):1017–1021. https://doi.org/10.1001/archneur.57.7.1017 PMID: 10891984

15. Yoon B, Shim YS, Lee KS, Shon YM, Yang DW: Region-specific changes of cerebral white matter during normal aging: a diffusion-tensor analysis. *Arch Gerontol Geriatr* 2008, 47(1):129–138. https://doi.org/10.1016/j.archger.2007.07.004 PMID: 17764763

16. Rathee R, Rallabandi VP, Roy PK: Age-Related Differences in White Matter Integrity in Healthy Human Brain: Evidence from Structural MRI and Diffusion Tensor Imaging. *Magn Reson Insights* 2016, 9:9–20. https://doi.org/10.4137/MRI.S39666 PMID: 27279747

17. Pfefferbaum A, Sullivan EV, Hedehus M, Lim KO, Adalsteinsson E, Moseley M: Age-related decline in brain white matter anisotropy measured with spatially corrected echo-planar diffusion tensor imaging. *Magn Reson Med* 2000, 44(2):259–268. https://doi.org/10.1002/1522-2594(200008)44:2<259::aid-mrm13>3.0.co;2-6 PMID: 10918325

18. Mandl RC, Schnack HG, Zwiers MP, Kahn RS, Hulshoff Pol HE: Functional diffusion tensor imaging at 3 Tesla. *Front Hum Neurosci* 2013, 7:817. https://doi.org/10.3389/fnhum.2013.00817 PMID: 24409133

19. Mandl RC, Schnack HG, Zwiers MP, van der Schaaf A, Kahn RS, Hulshoff Pol HE: Functional diffusion tensor imaging: measuring task-related fractional anisotropy changes in the human brain along white matter tracts. *PLoS One* 2008, 3(11):e3631. https://doi.org/10.1371/journal.pone.0003631 PMID: 18982065

20. Araque A, Navarrete M: Glial cells in neuronal network function. *Philos Trans R Soc Lond B Biol Sci* 2010, 365(1551):2375–2381. https://doi.org/10.1098/rstb.2009.0313 PMID: 20603358

21. Ogawa S, Lee TM, Kay AR, Tank DW: Brain magnetic resonance imaging with contrast dependent on blood oxygenation. *Proc Natl Acad Sci U S A* 1990, 87(24):9868–9872. https://doi.org/10.1073/pnas.87.24.9868 PMID: 2124706

22. Ostby I, Oyehaug L, Einevoll GT, Nagelhus EA, Plahte E, Zeuthen T, Lloyd CM, Ottersen OP, Omholt SW: Astrocytic mechanisms explaining neural-activity-induced shrinkage of extraneuronal space. *PLoS Comput Biol* 2009, 5(1):e1000272. https://doi.org/10.1371/journal.pcbi.1000272 PMID: 19165313

23. Dietzel I, Heinemann U, Lux HD: Relations between slow extracellular potential changes, glial potassium buffering, and electrolyte and cellular volume changes during neuronal hyperactivity in cat brain. *Glia* 1989, 2(1):25–44. https://doi.org/10.1002/glia.440020104 PMID: 2523337

24. Dietzel I, Heinemann U: Dynamic variations of the brain cell microenvironment in relation to neuronal hyperactivity. *Ann N Y Acad Sci* 1986, 481:72–86. https://doi.org/10.1111/j.1749-6632.1986.tb27140.x PMID: 3468867

25. Anderson AW, Zhong J, Petroff OA, Szafer A, Ransom BR, Prichard JW, Gore JC: Effects of osmotically driven cell volume changes on diffusion-weighted imaging of the rat optic nerve. *Magn Reson Med* 1996, 35(2):162–167. https://doi.org/10.1002/mrm.1910350206 PMID: 8622579

26. Rasmussen R, Nicholas E, Petersen NC, Dietz AG, Xu Q, Sun Q, Nedergaard M: Cortex-wide Changes in Extracellular Potassium Ions Parallel Brain State Transitions in Awake Behaving Mice. *Cell Rep* 2019, 28(5):1182–1194 e1184. https://doi.org/10.1016/j.celrep.2019.06.082 PMID: 31365863

27. Baylor DA, Nicholls JG: Changes in extracellular potassium concentration produced by neuronal activity in the central nervous system of the leech. *J Physiol* 1969, 203(3):555–569. https://doi.org/10.1113/jphysiol.1969.sp008879 PMID: 5387026

28. Xie L, Kang H, Xu Q, Chen MJ, Liao Y, Thiyagarajan M, O'Donnell J, Christensen DJ, Nicholson C, Iliff JJ et al: Sleep drives metabolite clearance from the adult brain. *Science* 2013, 342(6156):373–377. https://doi.org/10.1126/science.1241224 PMID: 24136970

29. Fjell AM, Westlye LT, Greve DN, Fischl B, Benner T, van der Kouwe AJ, Salat D, Bjornerud A, Due-Tonnessen P, Walhovd KB: The relationship between diffusion tensor imaging and volumetry as measures of white matter properties. *Neuroimage* 2008, 42(4):1654–1668. https://doi.org/10.1016/j.neuroimage.2008.06.005 PMID: 18620064

30. Tamnes CK, Ostby Y, Fjell AM, Westlye LT, Due-Tonnessen P, Walhovd KB: Brain maturation in adolescence and young adulthood: regional age-related changes in cortical thickness and white matter volume and microstructure. *Cereb Cortex* 2010, 20(3):534–548. https://doi.org/10.1093/cercor/bhp118 PMID: 19520764

31. Iliff JJ, Nedergaard M: Is there a cerebral lymphatic system? *Stroke* 2013, 44(6 Suppl 1):S93–95.

32. Iliff JJ, Lee H, Yu M, Feng T, Logan J, Nedergaard M, Benveniste H: Brain-wide pathway for waste clearance captured by contrast-enhanced MRI. *J Clin Invest* 2013, 123(3):1299–1309. https://doi.org/10.1172/JCI67677 PMID: 23434588

33. Rangroo Thrane V, Thrane AS, Plog BA, Thiyagarajan M, Iliff JJ, Deane R, Nagelhus EA, Nedergaard M: Paravascular microcirculation facilitates rapid lipid transport and astrocyte signaling in the brain. *Scientific reports* 2013, 3:2582. https://doi.org/10.1038/srep02582 PMID: 24002448

34. MacVicar BA, Newman EA: Astrocyte regulation of blood flow in the brain. *Cold Spring Harb Perspect Biol* 2015, 7(5).

35. Verkman AS, Binder DK, Bloch O, Auguste K, Papadopoulos MC: Three distinct roles of aquaporin-4 in brain function revealed by knockout mice. *Biochim Biophys Acta* 2006, 1758(8):1085–1093. https://doi.org/10.1016/j.bbamem.2006.02.018 PMID: 16564496

36. Gao X, Ming J, Liu S, Lai B, Fang F, Cang J: Sevoflurane enhanced the clearance of Abeta1-40 in hippocampus under surgery via up-regulating AQP-4 expression in astrocyte. *Life Sci* 2019, 221:143–151. https://doi.org/10.1016/j.lfs.2019.02.024 PMID: 30763576

37. Benveniste H, Lee H, Ding F, Sun Q, Al-Bizri E, Makaryus R, Probst S, Nedergaard M, Stein EA, Lu H: Anesthesia with Dexmedetomidine and Low-dose Isoflurane Increases Solute Transport via the Glymphatic Pathway in Rat Brain When Compared with High-dose Isoflurane. *Anesthesiology* 2017, 127 (6):976–988. https://doi.org/10.1097/ALN.0000000000001888 PMID: 28938276

38. Benveniste H, Heerdt PM, Fontes M, Rothman DL, Volkow ND: Glymphatic System Function in Relation to Anesthesia and Sleep States. *Anesth Analg* 2019, 128(4):747–758. https://doi.org/10.1213/ANE.0000000000004069 PMID: 30883420

39. Yokota H, Vijayasarathi A, Cekic M, Hirata Y, Linetsky M, Ho M, Kim W, Salamon N: Diagnostic Performance of Glymphatic System Evaluation Using Diffusion Tensor Imaging in Idiopathic Normal Pressure Hydrocephalus and Mimickers. *Curr Gerontol Geriatr Res* 2019, 2019:5675014. https://doi.org/10.1155/2019/5675014 PMID: 31320896

40. DiNuzzo M, Nedergaard M: Brain energetics during the sleep-wake cycle. *Curr Opin Neurobiol* 2017, 47:65–72. https://doi.org/10.1016/j.conb.2017.09.010 PMID: 29024871

41. Conde C, Caceres A: Microtubule assembly, organization and dynamics in axons and dendrites. *Nat Rev Neurosci* 2009, 10(5):319–332. https://doi.org/10.1038/nrn2631 PMID: 19377501

42. Lasser M, Tiber J, Lowery LA: The Role of the Microtubule Cytoskeleton in Neurodevelopmental Disorders. *Front Cell Neurosci* 2018, 12:165. https://doi.org/10.3389/fncel.2018.00165 PMID: 29962938

43. Kohtala S, Theilmann W, Suomi T, Wigren HK, Porkka-Heiskanen T, Elo LL, Rokka A, Rantamaki T: Brief Isoflurane Anesthesia Produces Prominent Phosphoproteomic Changes in the Adult Mouse Hippocampus. *ACS Chem Neurosci* 2016, 7(6):749–756. https://doi.org/10.1021/acschemneuro.6b00002 PMID: 27074656

44. Le Freche H, Brouillette J, Fernandez-Gomez FJ, Patin P, Caillierez R, Zommer N, Sergeant N, Buee-Scherrer V, Lebuffe G, Blum D et al: Tau phosphorylation and sevoflurane anesthesia: an association

to postoperative cognitive impairment. *Anesthesiology* 2012, 116(4):779–787. https://doi.org/10.1097/ALN.0b013e31824be8c7 PMID: 22343471

45. Jiang J, Jiang H: Effect of the inhaled anesthetics isoflurane, sevoflurane and desflurane on the neuropathogenesis of Alzheimer's disease (review). *Mol Med Rep* 2015, 12(1):3–12. https://doi.org/10.3892/mmr.2015.3424 PMID: 25738734

46. Tao G, Zhang J, Zhang L, Dong Y, Yu B, Crosby G, Culley DJ, Zhang Y, Xie Z: Sevoflurane induces tau phosphorylation and glycogen synthase kinase 3beta activation in young mice. *Anesthesiology* 2014, 121(3):510–527. https://doi.org/10.1097/ALN.0000000000000278 PMID: 24787352

47. Jevtovic-Todorovic V, Absalom AR, Blomgren K, Brambrink A, Crosby G, Culley DJ, Fiskum G, Giffard RG, Herold KF, Loepke AW et al: Anaesthetic neurotoxicity and neuroplasticity: an expert group report and statement based on the BJA Salzburg Seminar. *British journal of anaesthesia* 2013, 111(2):143–151. https://doi.org/10.1093/bja/aet177 PMID: 23722106

48. Planel E, Bretteville A, Liu L, Virag L, Du AL, Yu WH, Dickson DW, Whittington RA, Duff KE: Acceleration and persistence of neurofibrillary pathology in a mouse model of tauopathy following anesthesia. *FASEB J* 2009, 23(8):2595–2604. https://doi.org/10.1096/fj.08-122424 PMID: 19279139

49. Schettini A, Furniss WW: Brain water and electrolyte distribution during the inhalation of halothane. *British journal of anaesthesia* 1979, 51(12):1117–1124. https://doi.org/10.1093/bja/51.12.1117 PMID: 526377

50. Murr R, Berger S, Schurer L, Peter K, Baethmann A: Influence of isoflurane, fentanyl, thiopental, and alpha-chloralose on formation of brain edema resulting from a focal cryogenic lesion. *Anesth Analg* 1995, 80(6):1108–1115. https://doi.org/10.1097/00000539-199506000-00007 PMID: 7762836

51. Larach DR, Schuler HG: Direct vasodilation by sevoflurane, isoflurane, and halothane alters coronary flow reserve in the isolated rat heart. *Anesthesiology* 1991, 75(2):268–278. https://doi.org/10.1097/00000542-199108000-00015 PMID: 1859014

52. Iida H, Ohata H, Iida M, Watanabe Y, Dohi S: Isoflurane and sevoflurane induce vasodilation of cerebral vessels via ATP-sensitive K+ channel activation. *Anesthesiology* 1998, 89(4):954–960. https://doi.org/10.1097/00000542-199810000-00020 PMID: 9778013

53. Scheller MS, Tateishi A, Drummond JC, Zornow MH: The effects of sevoflurane on cerebral blood flow, cerebral metabolic rate for oxygen, intracranial pressure, and the electroencephalogram are similar to those of isoflurane in the rabbit. *Anesthesiology* 1988, 68(4):548–551. https://doi.org/10.1097/00000542-198804000-00012 PMID: 3354892

54. Goren S, Kahveci N, Alkan T, Goren B, Korfali E: The effects of sevoflurane and isoflurane on intracranial pressure and cerebral perfusion pressure after diffuse brain injury in rats. *J Neurosurg Anesthesiol* 2001, 13(2):113–119. https://doi.org/10.1097/00008506-200104000-00008 PMID: 11294452

55. Takahashi H, Murata K, Ikeda K: Sevoflurane does not increase intracranial pressure in hyperventilated dogs. *British journal of anaesthesia* 1993, 71(4):551–555. https://doi.org/10.1093/bja/71.4.551 PMID: 8260306

56. Kotani J, Sugioka S, Momota Y, Ueda Y: Effect of sevoflurane on intracranial pressure, sagittal sinus pressure, and the intracranial volume-pressure relation in cats. *J Neurosurg Anesthesiol* 1992, 4(3):194–198. https://doi.org/10.1097/00008506-199207000-00008 PMID: 15815463

57. Gideon P, Sorensen PS, Thomsen C, Stahlberg F, Gjerris F, Henriksen O: Increased brain water self-diffusion in patients with idiopathic intracranial hypertension. *AJNR Am J Neuroradiol* 1995, 16(2):381–387. PMID: 7726088

58. Ulug AM, Truong TN, Filippi CG, Chun T, Lee JK, Yang C, Souweidane MM, Zimmerman RD: Diffusion imaging in obstructive hydrocephalus. *AJNR Am J Neuroradiol* 2003, 24(6):1171–1176. PMID: 12812949

59. Dorenbeck U, Schlaier J, Feuerbach S, Seitz J: [Diffusion-weighted imaging in the diagnostic evaluation of the hydrocephalus in patients with acute or chronic increase in cerebral pressure]. *RoFo: Fortschritte auf dem Gebiete der Rontgenstrahlen und der Nuklearmedizin* 2005, 177(1):99–104. https://doi.org/10.1055/s-2004-813728 PMID: 15657827

60. Casaca-Carreira J, Temel Y, Hescham SA, Jahanshahi A: Transependymal Cerebrospinal Fluid Flow: Opportunity for Drug Delivery? *Mol Neurobiol* 2017. https://doi.org/10.1007/s12035-017-0501-y PMID: 28455692

61. Zhao C, Li Y, Cao W, Xiang K, Zhang H, Yang J, Gan Y: Diffusion tensor imaging detects early brain microstructure changes before and after ventriculoperitoneal shunt in children with high intracranial pressure hydrocephalus. *Medicine (Baltimore)* 2016, 95(42):e5063.

62. Hoffmann J, Kreutz KM, Csapo-Schmidt C, Becker N, Kunte H, Fekonja LS, Jadan A, Wiener E: The effect of CSF drain on the optic nerve in idiopathic intracranial hypertension. *J Headache Pain* 2019, 20(1):59. https://doi.org/10.1186/s10194-019-1004-1 PMID: 31122204

63. Assaf Y, Ben-Sira L, Constantini S, Chang LC, Beni-Adani L: Diffusion tensor imaging in hydrocephalus: initial experience. *AJNR Am J Neuroradiol* 2006, 27(8):1717–1724. PMID: 16971621

64. Sarica A, Curcio M, Rapisarda L, Cerasa A, Quattrone A, Bono F: Periventricular white matter changes in idiopathic intracranial hypertension. *Ann Clin Transl Neurol* 2019, 6(2):233–242. https://doi.org/10.1002/acn3.685 PMID: 30847356
